# Supplementary material for: Genetic insights into Cyphocharax magdalenae (Characiformes: Curimatidae): Microsatellite loci development and population analysis in the Cauca River, Colombia
Source: PLoS One. 2024 Apr 16;19(4):e0302273. doi: 10.1371/journal.pone.0302273 (PMC11020439; doi:10.1371/journal.pone.0302273)
Supplement: Attachment — Submitted filename: Cyphocharax_magdalenae_Ochoa-Aristizabal&Marquez_KLGC.docx [file pone.0302273.s004.docx]

**Genetic insights into *Cyphocharax magdalenae* (Characiformes: Curimatidae): microsatellite loci development and population analysis in the Cauca River, Colombia**

Ana Maria Ochoa-Aristizábal^1^ ^¶^, Edna Judith Márquez^1^ ^¶*^

^1^Laboratorio de Biología Molecular y Celular, Escuela de Biociencias, Facultad de Ciencias Universidad Nacional de Colombia – Sede Medellín, Medellín, Antioquia, Colombia.

* Corresponding author

ejmarque@unal.edu.co (EJM)

^¶^ These authors contributed equally to this work

**Abstract**

*Cyphocharax magdalenae*, a Colombian freshwater fish species, plays a vital role in nutrients distribution and serves as a significant food source for other fish species and local fishing communities. Considered a short-distance migratory species, *C. magdalenae* populations face substantial risks due to human activities impacting their habitats. To address the lack of knowledge regarding genetic diversity and population structure, this study employed next-generation sequencing technology to develop species-specific microsatellite loci and conducted a population genetics analysis of *C. magdalenae* in the middle and lower sectors of Cauca River, Colombia. Out of 30 pairs of microsatellite primers evaluated in 324 individuals; 14 *loci* were polymorphic and in agreement with Hardy-Weinberg equilibrium in at least on population, showing linkage equilibrium. The results showed high levels of genetic diversity compared to other neotropical Characiformes, with inbreeding coefficients similar to those reported for other phylogenetically close species. Moreover, *C. magdalenae* exhibits seasonal population structure (rainy-dry) consisting of two genetic stocks showing bottleneck signals and high effective population numbers. This information is fundamental for understanding of the current genetics of the species and future construction of management programs of this fishery resource and for the.

**Introduction**

Curimatidae family encompasses 8 genera and 119 valid species distributed in South America (Fricke et al., 2023). *Cyphocharax* Fowler 1906 genus is one of the most abundant having 47 valid species (Fricke et al., 2023) and wide distribution ranging from the Pacific rivers in southern Costa Rica to the La Plata river and various coastal drainages in central Argentina (Maldonado-Ocampo et al., 2008; Melo et al., 2018). It has been found that Atlantic basins have a much more diverse group expanding from the Orinoco river, the Amazonas river, rivers from the Guayanas and eastern Brazil; on the contrary, a less diverse group of species habits the rivers in northern Peru to Costa Rica, comprising the tributaries of Magdalena and Maracaibo (Vari et al., 2010).

*Cyphocharax magdalenae* (Steindachner 1878) commonly known as “viejito” or “Yalúa” distributes from the Coto river in southeastern Cosa Rica (Angulo et al., 2013), Panamá (Smith et al., 2004; Vega et al., 2006), to the Maracaibo lake and its tributaries in Venezuela (Vari, 1992) and Colombia (DoNascimiento et al., 2017). In Colombia, *C. magdalenae* is the only species of the genus reported for the Atrato, Sinú, Canalete and Magdalena-Cauca basin (Lasso et al., 2011; DoNascimiento et al., 2017). It represents the 30% in number of individuals within the floodplains of lower Magdalena-Cauca; it participates in transportation of energy, nutrients, coal, minerals and sediment resuspension in the Cauca basin **(Jimenéz-Segura & Lasso, 2020). It has short-distance migration (<100 km) (Zapata & Usma, 2013) and it is potamodromous, i.e., a purely freshwater fish; its migrations are associated to reproductive purposes, having two spawns per year (Lasso et al., 2011; Jimenéz-Segura & Lasso, 2020). Despite the information on several reproductive aspects is scarce** (Díaz Ortíz et al., 2021)**, it is known that it does not build nests, nor show parental care of embryos, can have two spawns a year with small (0.73 ± 0.04 mm), adhesive and with small perivitelline space eggs** (Lasso et al., 2011; Atencio-García et al., 2022) **and its generational time is 2.6 years** (Puentes et al., 2014).

*Cyphocharax magdalenae* **is listed as a Least Concern species by The International Union for Conservation of Nature (IUCN). It has been gaining fishing importance during the last years due to the replacement of captured traditional species such as** catfish *Prochilodus magdalenae* Steindachner 1879 and Blanquillo *Sorubim cuspicaudus* Littmann, Burr & Nass 2000 **(Blanco et al., 2005; Jimenéz-Segura & Lasso, 2020). Nevertheless, its notable mortality because of overfishing suggests a possible decrease in its populations (Lasso et al., 2011; Duarte et al., 2019, 2021).**

The latter is relevant since currently ***C. magdalenae* lacks genetic studies that allow knowing the present status of the species and delving into its ecological processes, which hinders the development and application of plans for the protection and conservation of the species. This scarcity in information contrasts with population genetic studies performed for *Curimata mivartii* Steindachner 1878 (Landínez-García & Marquez, 2018) and species with fishing relevance of the phylogenetically close family Prochilodontidae like *Prochilodus magdalenae* (Aguirre-Pabón et al., 2013; Berdugo & Barandica, 2014; Landínez-García et al., 2020),** ***Prochilodus reticulatus* Valenciennes 1850 (Macias et al., 2009) and *Ichthyoelephas longirostris* (Steindachner 1879) (Landínez-García & Marquez, 2016); in addition to species of other families habiting the Magdalena-Cauca basin such as *Pseudoplatystoma magdaleniatum* Buitrago-Suárez & Burr 2007 (Gallo & Díaz-sarmiento, 2003; García-Castro et al., 2021), *Brycon henni* Eigenmann 1913 (Pineda et al., 2007; Hurtado-Alarcón et al., 2011; Landínez-García & Márquez, 2020), *S. cuspicaudus, Ageneiosus pardalis* Lütken 1874*, Pimelodus grosskopfii* Steindachner 1879 (Restrepo-Escobar & Márquez, 2020), *Pimelodus yuma* Villa-Navarro & Acero P. 2017 (Joya et al., 2021), *Pseudopimelodus magnus* Restrepo-Gómez, Rangel-Medrano, Márquez & Ortega-Lara 2020 and *Pseudopimelodus atricaudus* Restrepo-Gómez, Rangel-Medrano, Márquez & Ortega-Lara 2020 (Rangel-Medrano & Márquez, 2021). Among the different markers used in the cited studies, microsatellites stand out as the most used tool to solve questions at micro-evolutive scale in population genetics.**

**To estimate the genetic status of the species in the lower sector of the Cauca river, this study contrasted three hypotheses: (i) *C. magdalenae* shows signs of genetic deterioration with low levels of genetic variability, high levels of consanguinity and recent bottlenecks as a result of habitat alterations due to different anthropogenic activities; (ii) the species exhibit gene flow as a result of the scarce or non-existent geographic barriers in this habit, which is based on evidence found for other Curimatidae species (Landínez-García & Marquez, 2018); and (iii) the climatic seasons that influence fish migration in the Cauca river structure the population of *C. magdalenae.* For contrasting these hypotheses, species-specific microsatellite loci were identified de novo and characterized from low-coverage genome sequencing of an individual of *C. magdalenae* through next generation technology Illumina Miseq v2. Moreover, polymorphism levels and genetic structure exhibited by the species in the lower sector of the Cauca river were assessed following the methodology described by Landínez-García & Márquez in 2016 and 2018. The Cauca river, main tributary of the Magdalena-Cauca basin (González-López & Carvajal-Escobar, 2020; Cerón et al., 2021), has great economic importance because of its influence in most prioritized productive chains in Colombia, which include small exploitations, extensive industries, monocultures, energy generation, mineral extraction, agriculture and fishing (Carvajal et al., 2016; González-López & Carvajal-Escobar, 2020; Jimenéz-Segura & Lasso, 2020). This has caused that fishes are one of the most threatened biological resources, with little- or unknown local priorities over the rivers (Corporación Autónoma Regional de Cundinamarca (CAR), 2012; Hernández-Barrero et al., 2021).**

****Materials and Methods****

****Biological material and study area****

This study analyzed a total of 324 muscle tissues and/or fins of *C. magdalenae* preserved in ethanol 70%, provided between 2019 and 2021 by Universidad de Antioquia, Universidad de Córdoba, and Universidad Nacional de Colombia Sede Medellín, through scientific cooperation agreement CT-2019-000661, framed within the environmental license # 0155 of 30 January 2009 of Ministerio de Ambiente, Vivienda y Desarrollo Territorial for the construction of hydroelectric Ituango. Samples from individuals were collected in 19 sites of the main channel, some swamps, and tributaries of the lower sector of the Cauca river. This zone, characterized by a large alluvial plain surrounded by mountains and flat and wavy surfaces wherein various swampy complexes are formed (Mejía et al., 2007; Betancur et al., 2009), has been exposed to different anthropic activities including stockbreeding, fishing and extensive cultures (Corantioquia - Universidad de Antioquia, 2014). Additionally, high levels of mercury and sediments are reported, as well as elevated amounts of vegetations in the main courses of the river as a result of deforestation and flooding that may affect the ichthyofauna (Corantioquia, 2016; Cruz-Esquivel & Marrugo-Negrete, 2022). According to their distribution, samples were grouped in five sectors (**Fig 1**) previously described by Landínez-García & Márquez (2016): S4 (Jardín and Man river), S5 (Margento, Nechí river, Palanca, La Trinidad stream and swamps La Ilusión and Palomar), S6 (La Encaramada and swamps La Caimanera, Ciritongo, Culebras and Grande), S7 (Tres cruces and swamps El Floral, Nueva and Santa Catalina) and S8 (Punta Cartagena and swamp Piqué).

**Fig 1.** **Sampling sites (stars) of *Cyphocharax magdalenae* in the low section of the Cauca river (S4-S8).**

**Microsatellite *loci* identification and development of primers**

Methodology described by Landínez-García & Márquez (2016, 2018) was followed for developing species-specific microsatellite loci for *C. magdalenae.* The genomic library was built from total DNA extraction from the spin (5,775ug) of a *C. magdalenae* individual captured in the Cauca river (CYPHO11886) using the DNA extraction reagents and recommendations of the manufacturer PureLink genomic DNA Mini Kit (Invitrogen), then it was used for NGS surface sequencing (Whole Genome Sequencing), through Illumina Miseq (300 PE). After cleaning the raw reads using PRINSEQ lite (removal of adapter sequences utilizing <Q30 quality bases), 100,000 high-quality extended reads were analyzed with PAL_FINDER v0.02.03 (Castoe et al., 2010) for extracting reads containing tri-, tetra- and pentanucleotide microsatellite motifs. Then, Primer3 v2.0 (Rozen & Skaletsky, 2000) was used for the primer designs in the flanking sequences of the microsatellite *loci* and ultimately the correct evaluation of the primers was tested with ePCR (Rotmistrovsky et al., 2004). Selection of the set of the microsatellite loci was performed according to already stablished features for validating new primers of microsatellite *loci* (Fernandez-Silva et al., 2013; Schoebel et al., 2013).

For genotyping, 10μl reaction mixtures were used with final concentrations of 0.3pmol/μl of each forward primer tagged on the 5’ end with one of the adapters (tails A, B, C and D) described by Blacket et al., 2012, 6pmol/μl of each reverse primer, 0.5pmol/μl fluorescently labeled adapter (6-FAM, VIC, NED and PET, Applied Biosystems), 1X Master Mix, 2.5% v/v GC Enhancer Platinum Multiplex PCR Master Mix (Applied Biosystems) and 3-5μg/μl DNA isolated using the Purelink® purification kit (Thermo Scientific) with a modification in digestion time of 24 h when working with fins. Thermal profiles included an initial denaturalization step of 90 °C for 35 s and an annealing step of 56 °C for 35 s (with no final elongation). Subsequently, amplicons were separated by electrophoresis on an ABI 3730 XL automated sequencer using 600LIZ as the internal molecular size. Then, GeneMarker® v3.0.0 was employed to denote the allele fragments according to their molecular size (100 to 450 pb) and Micro-Checker v.2.2.3 (Van Oosterhout et al., 2004) was run to detect potential genotyping errors.

**Polymorphism, genetic diversity, atypical *loci* detection, and demographical events**

Polymorphism information content (PIC) and the average number of alleles per *locus* for each microsatellite marker were calculated using Cervus v3.0.7 (Marshall et al., 1998) and GenAlEx v6.5.03 (Peakall & Smouse, 2006). Estimation of observed (H_O_) and expected (H_E_) heterozygosities, consanguinity coefficient (F_IS_) and tests for departures from Hardy-Weinberg equilibrium were performed using Arlequin v3.5.2.2 (Excoffier et al., 2005), applying the sequential Bonferroni correction for the statistical significance in the multiple comparisons (Holm, 1979; Rice, 1989).

For detecting atypical *loci* and determining evolutive forces acting over the microsatellite *loci*, BayeScan v2.1 (Foll & Gaggiotti, 2008) was used for performing an analysis employing the parameters for prior odds of 10:1 for the neutral model, 20 pilot runs, each having 5,000 iterations, followed by 100,000 iterations and a burn-in of 50,000. Critical values were based on the posterior probability of the Bayes factor, following the Jeffrey’s scale (Nagin, 1999), which sets a probability of 0.76 as substantial evidence for selection.

To determine recent genetic bottlenecks, excess heterozygosity was tested under the mutation drift equilibrium assumption in three mutational models of the microsatellite loci (IAM: infinite alleles model, SMM: stepwise mutational model, TPM: two-phase model) through Wilcoxon signed rank test (Luikart & Cornuet, 1998) included in Bottleneck v.1.2.02 (Piry et al., 1999). Moreover, M ratio (mean ratio of the number of alleles to allele size range; Garza & Williamson, 2001) included in Arlequin v3.5.2.2 (Excoffier et al., 2005) was calculated following the criteria according to which values lower than 0.68 indicate recent and severe reductions in population size (Garza & Williamson, 2001). Furthermore, effective population size (N_e_) of the species was determined in each of the assessed populations using the linkage disequilibrium method and a minimum allele frequency of 0.02 implemented in NeEstimator v2.1 (Do et al., 2014). Change in H_E_ of the species in 10, 50 and 100 generations (t) was estimated based on the equation proposed by Crow & Kimura, 1970 ($Ht/H0=\left[ 1-1/\left( 2Ne \right) \right]^{t}$). Genetic deterioration grade was stablished using the critical values of 25% Ht reduction in 10 (critically in danger), 50 (in danger) and 100 (vulnerable) generations (Willoughby et al., 2015).

**Genetic structure**

Bayesian analysis of population partitioning in STRUCTURE v.2.3.4 (Pritchard et al., 2000) was performed to determine samples grouping according to their co-ancestry coefficient. Parameters included 1,000,000 Monte Carlo Markov steps; 100,000 iterations were dismissed as burn-in to estimate each K value (1 – 8). Each analysis was repeated 20 times. For a best estimation of genetic stocks (K), STRUCTURESELECTOR (Li & Liu, 2018), was used to calculate the ΔK ad hoc statistic (Evanno et al., 2005), estimators MEDMEANK, MAXMEANK, MEDMEDK and MAXMEDK (Puechmaille, 2016), and to graphically represent the results using the integrated CLUMPAK software (Kopelman et al., 2015).

Genetic differentiation of *C. magdalenae* among sectors, genetic groups suggested by STRUCTURE and seasons (rainy and dry) was calculated using the standardized statistics F_ST_ (Wright, 1943, 1965; Meirmans, 2006) and Jost’s D_EST_ (Jost, 2008; Meirmans & Hedrick, 2011), and analysis of molecular variance (AMOVA) ) (Meirmans, 2006) using GenAlEx v6.503 software (Peakall & Smouse, 2006). Additionally, a discriminant analysis of principal components (DAPC) was performed in polymorphic loci genotype resulting in 324 analyzed individuals using the R- package Adegenet (Jombart, 2008). Moreover, allele frequency distributions in genetic stocks were tested by a G-test in Genepop v. 4.7.5. software (Rousset, 2008).

**Results**

**Identification of microsatellite *loci* and detection of atypical *loci***

Low-coverage genome sequencing raw data provided a total of 3.224.415.544 base pairs (bp), 10.712.344 reads, 41,28 %GC and 83,72 % Q30. After depuration, 100.000 reads (42.621.284 bp) were obtained, 37.119 out of which had compound repeat motifs, 1.054 interrupted repeat motifs and 71.376 contained microsatellite *loci* with direct repetitions (53.649 potentially amplifiable *loci*). Furthermore, 30.120 were validated using electronic PCR.

A group of 16 out of 30 tested *loci* showed clearly defined peaks and absence of stutter bands in the electropherograms; these microsatellite loci include tri- (3) and tetra- (13) nucleotides motifs. Micro-Checker analysis did not indicate stutter associated genotyping errors nor an allele loss in the *loci* (dropout) and linkage disequilibrium tests were not significant. Departures of allele frequencies from Hardy-Weinberg equilibrium were identified as well as heterozygote deficits (**Table 1**). In general, microsatellite *loci* exhibited allele size between 106 and 414 bp with PIC values ranging from 0.733 to 0.954 and H_O_ and H_E_ between 0.408-0.916 and 0.762-0.957, respectively (**Table 1**); no evidence of atypical *loci* was detected by Bayescan. Nonetheless, null alleles evidence was found in *loci* Cym10 and Cym16, for which they were excluded in subsequent analysis.

**Table 1.** **Primer sequences and features of 16 microsatellite *loci* selected for *Cyphocharax magdalenae*.**

| Locus | Motif | Primer sequence (5’ – 3’) | Range (bp) | | Na | PIC | H_O_ | H_E_ | P_HWE_ |
| --- | --- | --- | --- | --- | --- | --- | --- | --- | --- |
| Cym03 | (AAAG)n | F:TGCACTTAAATCCTGTCCATAAGC R:TCTGGATTGAGGCTTGAACG | 212-264 | 13 | | 0.733 | 0.716 | 0.762 | 0.000* |
| Cym04 | (ATCT)n | F:TGAGATTCATAAATTGGGAAGAGG R:AAAGGCATGTCTGTGTTGCC | 230-298 | 18 | | 0.898 | 0.840 | 0.908 | 0.010* |
| Cym06 | (AAAT)n | F:TCATCTTCCATTATTTGTGCTGG R:GAGGAACGATTTACCCATGC | 224-324 | 23 | | 0.920 | 0.819 | 0.926 | 0.000* |
| Cym07 | (ATT)n | F:AGGCTCATTAGTACCAGCAGC R:TGTGCACAACAGGAACATCC | 111-195 | 27 | | 0.941 | 0.861 | 0.945 | 0.000* |
| Cym08 | (ATAC)n | F:ACCAAACTGGAAGACAGCCC R:TAAACAACTTCCACACCGGC | 216-332 | 29 | | 0.939 | 0.765 | 0.943 | 0.000* |
| Cym10 | (TTC)n | F:TTACTGCGAGCGGACCTACC R:CAGCCAACATGGTTTGTATGG | 236-407 | 40 | | 0.929 | 0.647 | 0.935 | 0.000* |
| Cym11 | (AAAT)n | F:AACCCAAGCCTTCAATCAGG R:TGACCAACCGTATCCCTGC | 280-312 | 9 | | 0.771 | 0.691 | 0.797 | 0.000* |
| Cym14 | (ATCT)n | F:TGTATGGTTTTGTCATTTGCACC R:CCTCAGGGAGTTATTTGCCG | 215-375 | 35 | | 0.954 | 0.842 | 0.957 | 0.000* |
| Cym16 | (ATCT)n | F:GAAAGGGCAAATAACCTACTGC R:ATACCGGTTGTGCTGTGACC | 254-414 | 34 | | 0.928 | 0.408 | 0.934 | 0.000* |
| Cym18 | (ATGG)n | F:CAGGGCAAATACTGCCTTCC R:TCCACAAAGAAGGCCACTCC | 185-289 | 27 | | 0.922 | 0.873 | 0.928 | 0.023* |
| Cym19 | (TCTG)n | F:TGGCGTATAAACATCAGCGG R:CTGGAACTGCCAAAGATCCC | 157-225 | 17 | | 0.846 | 0.851 | 0.861 | 0.530 |
| Cym24 | (ATT)n | F:GCTGGACTTCCATATCCACTCG R:GCTATTTCTTCCCCTGCATCG | 250-325 | 26 | | 0.946 | 0.910 | 0.950 | 0.011* |
| Cym25 | (AAAT)n | F:CCCTGCCTTCATTAGCATCC R:CCAGCCTTCCTCTTTCTCCC | 106-198 | 23 | | 0.910 | 0.843 | 0.918 | 0.000* |
| Cym26 | (AAAG)n | F:CAACTCGGAGTGACCTACCG R:CTGTGTGGACCGAGTGTTCC | 125-197 | 19 | | 0.900 | 0.833 | 0.909 | 0.000* |
| Cym29 | (AATG)n | F:ATCTGGCAGTTTGTCCAGGG R:CAGTGTCCCAGACCGAGACC | 190-234 | 12 | | 0.791 | 0.792 | 0.816 | 0.114 |
| Cym30 | (AGTG)n | F:GACGTTCCATCGCTTCATCC R:GAGAGAGTCTGACTGATGACCAGC | 184-236 | 14 | | 0.751 | 0.763 | 0.782 | 0.600 |

Na number of alleles per locus; PIC polymorphic information content; H_O_ Observed heterozygosity; H_E_ Expected heterozygosity; P_HWE_ P value of tests for departure from Hardy-Weinberg equilibrium (α: 0.05); * Statistical significance.

**Genetic diversity and demographic events**

Genetic diversity estimators (**Table 2**) showed that S7 had the lower average number of alleles per *locus* (15.714 alleles/*locus*) while S5 exhibited the higher mean number (18.857 alleles/*locus*). Mean H_O_ was lower in S7 (0.772) and higher in S8 (0.827) and mean H_E_ displayed the lower value in S5 (0.880) and the higher in S4 (0.887). Only two *loci* (Cym8 and Cym14) out of the 14 selected departed from the Hardy-Weinberg equilibrium in four of the five evaluated sites and the remaining fulfilled the assumption in at least two sites. A similar result of genetic diversity was observed in the genetic stocks (Stock1 and Stock2, **Table 2**) found by the genetic structure Bayesian analysis, as shown below.

Moreover, multiloci values (across *loci*) of the inbreeding coefficient for each of the populations showed statistical significance with greater impact in population S7 (F_IS_=0.120, P=0.000). These values, although remained significant, significantly decreased in the stocks (F_IS_Stock1=0.051, P=0.000; F_IS_Stock2=0.071, P=0.000).

**Table 2. Genetic diversity of *Cyphocharax magdalenae* in five sections (S4, S5, S6, S7 and S8) of the Cauca river in Colombia.**

| Pop |  | *Cym19* | *Cym 4* | *Cym3* | *Cym29* | *Cym25* | *Cym7* | *Cym24* | *Cym14* | *Cym8* | *Cym30* | *Cym11* | *Cym6* | *Cym18* | *Cym26* | Across loci |
| --- | --- | --- | --- | --- | --- | --- | --- | --- | --- | --- | --- | --- | --- | --- | --- | --- |
| S4 | Na | 14 | 15 | 11 | 9 | 19 | 22 | 24 | 27 | 21 | 11 | 8 | 19 | 19 | 14 | 16.643 |
|  | H_O_ | 0.956 | 0.822 | 0.773 | 0.778 | 0.711 | 0.800 | 0.886 | 0.841 | 0.791 | 0.844 | 0.667 | 0.795 | 0.800 | 0.889 | 0.811 |
|  | H_E_ | 0.878 | 0.910 | 0.756 | 0.801 | 0.920 | 0.947 | 0.950 | 0.957 | 0.924 | 0.781 | 0.830 | 0.903 | 0.926 | 0.912 | 0.885 |
|  | P_HWE_ | 0.584 | 0.269 | 0.117 | 0.019* | 0.000* | 0.004* | 0.069 | 0.002* | 0.147 | 0.854 | 0.010* | 0.131 | 0.080 | 0.195 | 0.000* |
|  | F_IS_ | -0.090 | 0.098 | -0.023 | 0.029 | 0.229 | 0.157 | 0.068 | 0.123 | 0.145 | -0.083 | 0.199 | 0.120 | 0.137 | 0.026 | 0.076* |
| S5 | Na | 14 | 15 | 12 | 9 | 22 | 26 | 24 | 32 | 27 | 12 | 9 | 20 | 24 | 18 | 18.857 |
|  | H_O_ | 0.796 | 0.860 | 0.652 | 0.811 | 0.936 | 0.849 | 0.926 | 0.883 | 0.766 | 0.772 | 0.702 | 0.796 | 0.883 | 0.777 | 0.815 |
|  | H_E_ | 0.844 | 0.913 | 0.695 | 0.803 | 0.929 | 0.943 | 0.947 | 0.958 | 0.951 | 0.792 | 0.819 | 0.917 | 0.907 | 0.900 | 0.880 |
|  | P_HWE_ | 0.613 | 0.467 | 0.273 | 0.696 | 0.237 | 0.083 | 0.626 | 0.000* | 0.000* | 0.444 | 0.000* | 0.001* | 0.066 | 0.004* | 0.000* |
|  | F_IS_ | 0.057 | 0.058 | 0.062 | -0.010 | -0.008 | 0.099 | 0.023 | 0.078 | 0.195 | 0.025 | 0.143 | 0.133 | 0.027 | 0.138 | 0.067* |
| S6 | Na | 14 | 15 | 10 | 9 | 18 | 25 | 25 | 31 | 25 | 12 | 9 | 20 | 24 | 16 | 18.071 |
|  | H_O_ | 0.883 | 0.853 | 0.750 | 0.753 | 0.844 | 0.896 | 0.948 | 0.792 | 0.787 | 0.727 | 0.727 | 0.805 | 0.883 | 0.882 | 0.824 |
|  | H_E_ | 0.875 | 0.903 | 0.788 | 0.814 | 0.913 | 0.939 | 0.953 | 0.957 | 0.941 | 0.785 | 0.772 | 0.926 | 0.941 | 0.909 | 0.887 |
|  | P_HWE_ | 0.698 | 0.019* | 0.306 | 0.116 | 0.006* | 0.091 | 0.039* | 0.000* | 0.004* | 0.208 | 0.000* | 0.004* | 0.172 | 0.131 | 0.000* |
|  | F_IS_ | -0.009 | 0.055 | 0.048 | 0.075 | 0.076 | 0.046 | 0.005 | 0.173 | 0.165 | 0.074 | 0.058 | 0.131 | 0.061 | 0.030 | 0.066* |
| S7 | Na | 12 | 15 | 11 | 10 | 15 | 23 | 21 | 24 | 20 | 10 | 9 | 17 | 18 | 15 | 15.714 |
|  | H_O_ | 0.865 | 0.806 | 0.811 | 0.806 | 0.730 | 0.811 | 0.778 | 0.838 | 0.622 | 0.722 | 0.568 | 0.806 | 0.865 | 0.784 | 0.772 |
|  | H_E_ | 0.848 | 0.917 | 0.794 | 0.815 | 0.894 | 0.947 | 0.944 | 0.950 | 0.933 | 0.779 | 0.790 | 0.933 | 0.915 | 0.923 | 0.884 |
|  | P_HWE_ | 0.129 | 0.064 | 0.743 | 0.412 | 0.062 | 0.001* | 0.001* | 0.163 | 0.000* | 0.297 | 0.001* | 0.022* | 0.053 | 0.061 | 0.000* |
|  | F_IS_ | -0.020 | 0.123 | -0.021 | 0.012 | 0.186 | 0.146 | 0.179 | 0.120 | 0.337 | 0.074 | 0.284 | 0.138 | 0.056 | 0.152 | 0.120* |
| S8 | Na | 16 | 18 | 12 | 11 | 21 | 23 | 23 | 29 | 23 | 12 | 7 | 20 | 25 | 16 | 18.286 |
|  | H_O_ | 0.817 | 0.829 | 0.676 | 0.814 | 0.859 | 0.901 | 0.930 | 0.845 | 0.800 | 0.761 | 0.718 | 0.887 | 0.901 | 0.845 | 0.827 |
|  | H_E_ | 0.866 | 0.898 | 0.788 | 0.820 | 0.904 | 0.940 | 0.951 | 0.953 | 0.950 | 0.773 | 0.722 | 0.929 | 0.936 | 0.903 | 0.881 |
|  | P_HWE_ | 0.416 | 0.102 | 0.001* | 0.854 | 0.019* | 0.041* | 0.786 | 0.000* | 0.000* | 0.737 | 0.719 | 0.436 | 0.519 | 0.007* | 0.000* |
|  | F_IS_ | 0.057 | 0.078 | 0.143 | 0.007 | 0.050 | 0.041 | 0.023 | 0.114 | 0.159 | 0.017 | 0.006 | 0.045 | 0.037 | 0.064 | 0.058* |
| Stock1 | Na | 15 | 15 | 12 | 9 | 22 | 25 | 25 | 33 | 26 | 13 | 9 | 19 | 25 | 16 | 18.857 |
|  | H_O_ | 0.895 | 0.856 | 0.696 | 0.768 | 0.860 | 0.850 | 0.956 | 0.886 | 0.805 | 0.723 | 0.702 | 0.830 | 0.886 | 0.816 | 0.823 |
|  | H_E_ | 0.861 | 0.899 | 0.740 | 0.783 | 0.893 | 0.939 | 0.942 | 0.956 | 0.945 | 0.774 | 0.785 | 0.906 | 0.926 | 0.896 | 0.875 |
|  | P_HWE_ | 0.796 | 0.764 | 0.046* | 0.524 | 0.099 | 0.353 | 0.960 | 0.000* | 0.002* | 0.204 | 0.000* | 0.157 | 0.031* | 0.013* | 0.000* |
|  | F_IS_ | -0.039 | 0.048 | 0.060 | 0.020 | 0.038 | 0.096 | -0.015 | 0.073 | 0.148 | 0.065 | 0.106 | 0.084 | 0.043 | 0.090 | 0.051* |
| Stock2 | Na | 17 | 18 | 12 | 12 | 23 | 26 | 24 | 32 | 29 | 14 | 7 | 23 | 26 | 19 | 20.143 |
|  | H_O_ | 0.828 | 0.832 | 0.726 | 0.806 | 0.833 | 0.867 | 0.885 | 0.818 | 0.743 | 0.785 | 0.686 | 0.813 | 0.867 | 0.842 | 0.809 |
|  | H_E_ | 0.860 | 0.910 | 0.773 | 0.815 | 0.896 | 0.942 | 0.948 | 0.956 | 0.941 | 0.786 | 0.696 | 0.930 | 0.928 | 0.888 | 0.876 |
|  | P_HWE_ | 0.905 | 0.028* | 0.026* | 0.280 | 0.078 | 0.070 | 0.023* | 0.000* | 0.000* | 0.839 | 0.771 | 0.001* | 0.003* | 0.061 | 0.000* |
|  | F_IS_ | 0.038 | 0.086 | 0.061 | 0.011 | 0.070 | 0.080 | 0.067 | 0.145 | 0.211 | 0.002 | 0.015 | 0.125 | 0.066 | 0.051 | 0.071* |

Na number of alleles per locus; H_O_ y H_E_ Observed and expected heterozygosities, respectively; F_IS_ inbreeding coefficient; P_HWE_ P statistical significance for the Hardy-Weinberg test (α: 0.05). * Statistical significance.

It was found that the assessed populations recently suffered a drastic reduction in population size since both the modified Garza-Williamson index (M-ratio: 0.238-0.256) and P values of the Wilcoxon signed rank test for the IAM and the TPM (except from one site) showed statistical significance (**Table 3**). Results for SMM, however, were not significant.

**Table 3. Bottleneck tests and effective population size (N_e_) of *Cyphocharax magdalenae* in the five sampling sites and genetic stocks of the Magdalena-Cauca basin. Statistical significance is noted in bold (P<0.05).**

| Pop | IAM^1^ | SMM^1^ | TPM^1^ | M-ratio^2^ | N_e_^3^ |
| --- | --- | --- | --- | --- | --- |
| S4 | 0.000* | 0.879 | 0.052 | 0.238 | 316 (166.4 – 1997.1) |
| S5 | 0.000* | 0.852 | 0.021* | 0.256 | ∞ (1194.3 - ∞) |
| S6 | 0.000* | 0.620 | 0.000* | 0.248 | 1869.2 (470.5 - ∞) |
| S7 | 0.000* | 0.932 | 0.015* | 0.243 | 223.9 (116.1 – 1571.6) |
| S8 | 0.000* | 0.966 | 0.039* | 0.250 | 233590.8 (692.8 - ∞) |
| Stock1 | 0.000* | 0.988 | 0.052 | 0.252 | 1464.6 (567.7 - ∞) |
| Stock2 | 0.000* | 0.979 | 0.002* | 0.257 | ∞ (2127 - ∞) |
| Overall | 0.000* | 0.891 | 0.000* | 0.262 | 2377.8 (1314.5 – 10252.9) |

^1^ P values of the Wilcoxon signed rank test of one tail in IAM, SMM and TPM; ^2^ M-ratio < 0.68 denote recent reductions in the population; ^3^ IC 95% using the JackKnife method (Waples & Do, 2008). * Statistical significance.

Furthermore, the effective size calculation in each of the sampled sites showed the lower values in S4 and S7 and the higher in S6 and S8; nevertheless, it was not possible to obtain a value for S5, and estimation was obtained only for one of the genetic stocks (Stock1) (**Table 3**). Due to the latter, in subsequent analysis the lower limits of confidence intervals were used for S5 and Stock2.

For all evaluated generations, the species is classified as non-threatened as no reduction percentage reached 25% (critical value of the classification) (**Table 4**). S8 showed the lower reduction percentage in all generations (0.002, 0.011, 0.021); nevertheless, S7 and S4 exhibited the greater loss of heterozygosity percentage in 100 generations with 20.034 and 14.645, respectively. Moreover, in the evaluation on genetic stocks no approximation surpassed the 5% reduction in all generations; in fact, similar values were found in each stock for each generation, although Stock1 showed slightly higher values.

**Table 4. Estimation of the change in H_E_ of *Cyphocharax magdalenae* in 10, 50 and 100 generations.**

| **Pop** | **t** | **H_t_ reduction** | **H_t_ reduction (%)** |
| --- | --- | --- | --- |
| S4 | 10 | 0.014 | 1.571 |
|  | 50 | 0.067 | 7.612 |
|  | 100 | 0.130 | 14.645 |
| S5 | 10 | 0.004 | 0.418 |
|  | 50 | 0.018 | 2.072 |
|  | 100 | 0.036 | 4.101 |
| S6 | 10 | 0.002 | 0.267 |
|  | 50 | 0.012 | 1.329 |
|  | 100 | 0.023 | 2.640 |
| S7 | 10 | 0.020 | 2.211 |
|  | 50 | 0.093 | 10.576 |
|  | 100 | 0.177 | 20.034 |
| S8 | 10 | 0.000 | 0.002 |
|  | 50 | 0.000 | 0.011 |
|  | 100 | 0.000 | 0.021 |
| Stock1 | 10 | 0.003 | 0.341 |
|  | 50 | 0.015 | 1.693 |
|  | 100 | 0.029 | 3.357 |
| Stock2 | 10 | 0.002 | 0.235 |
|  | 50 | 0.010 | 1.169 |
|  | 100 | 0.020 | 2.324 |

**Genetic structure**

In approximations set for determining population structure, it was found that the most backed group by all methods used in the Bayesian inference is a ΔK = 2. These two groups coexist in all sampled sites as confirmed by the STRUCTURE graphic (**Fig** **2A**). Additionally, it was observed that in sectors S4 and S5 predominates Stock1 (pink) while Stock2 (purple) is predominant in sectors S6, S7 and S8. This is corroborated by the discriminant analysis since it showed that the two genetic stocks were not homogeneously distributed (**Figs. 2B, 2C**); only one similarity was found in the distribution of the tested individuals in sites S6 and S7 (**Fig 2B**). Differences in the frequencies of the stocks agree with results obtained in AMOVA (F´_ST_= 0.003, P = 0.001) and the pairwise comparisons of the standardized statistics F’_ST_, D_EST_ (**Table 5**), which showed significant differences between S8 in comparison to the remaining sites, and S6 in relation to S4 and S5.

**Fig 2.** **Results of Bayesian inference of structure (a) and discriminant analysis of principal components of *Cyphocharax magdalenae*: (b) Five sites evaluated (c) Two genetic stocks.**

**Table 5. Pairwise comparisons of the genetic structure estimators of *Cyphocharax magdalenae*. Jost’s D_EST_ (upper diagonal) and F’_ST_ (below diagonal).**

| Pop | S4 | S5 | S6 | S7 | S8 |
| --- | --- | --- | --- | --- | --- |
| S4 | - | 0.000 | 0.031* | 0.014 | 0.082* |
| S5 | 0.005 | - | 0.019* | 0.007 | 0.042* |
| S6 | 0.007* | 0.004* | - | 0.000 | 0.020* |
| S7 | 0.008 | 0.006 | 0.006 | - | 0.037* |
| S8 | 0.010* | 0.006* | 0.005* | 0.008* | - |

* Statistical significance.

Genetic structure comparison by collect season (rainy: April-June, October-December; dry: January-March, July-August; Serna et al., 2021) and sampling years, in addition to confirming the coexisting of the two genetic stocks within the evaluated sectors of the Cauca river (ΔK=2, Mean LnP(K) = -22763.31000, MEDMEANK, MAXMEANK, MEDMEDK = 2; **Fig 3A**), revealed that during rainy seasons, the capture percentage was greater for Stock2 during the sampled years (2020: 91.7%; 2021: 94.3%), while Stock1 was primarily captured during dry season (2020: 67%; 2021: 58.8%) in comparison to the moderated captures of Stock2 (2020: 33%; 2021: 41.2%) (**Fig 3B**).

**Fig 3. Temporal structure of *Cyphocharax magdalenae* during 2019-2021 in the middle and lower sectors of the Cauca river (A) and percentage of each stock by season and year of capture (B). Rainy season (H): H_20: October 2020; H_21(1): April 2021; H_21(2): December 2021; Dry season (S): S_19: July-August-September 2019; S_20: February 2020; S_21: January 2021.**

**Discussion**

This study tested three hypotheses related to population genetics of *C. magdalenae*: (i) low levels of genetic variability, high levels of consanguinity and recent bottlenecks, probably as a result of alterations in its habitat due to different anthropogenic activities, (ii) gene flow because of scarce or non-existent geographic barriers of its habitat and (iii) seasonal population structure following the bimodal behavior of the Cauca river. To contrast these hypotheses, this study developed a set of 30 microsatellite loci of neotropical fish *C. magdalenae* of Colombia; 16 out of which having tri- and tetranucleotide repetition motifs showed to be polymorphic, highly informative and with ability to detect diversity levels and genetic structure in the low section of the Cauca river.

The selected microsatellite loci were preferably those having tri- and tetranucleotide repetition motifs as these repetition patterns are the most recommended due to their simplicity for genotyping and allele classification (Castoe et al., 2010, 2012; Guichoux et al., 2011). All microsatellite *loci* exhibited PIC values that allow describing them as highly informative according to Bostein et al., 1980; moreover, these values were higher than those reported for *C. mivartii* (0.549 – 0.946), *I. longirostris* (0.375 – 0.871) and *P. magdalenae* (0.399 – 0.949) (Landínez-García & Marquez, 2016, 2018; Landínez-García et al., 2020).

Results obtained in the genetic analysis of 324 samples of *C. magdalenae* do not support the hypothesis of low levels of genetic variability in the species as it showed high mean values of alleles/*locus* (sampling site: 17.514, Stock: 19.500) and H_E_ (sampling site: 0.883 – Stock: 0.876) in relation to those reported for neotropical Characiformes (Na: 10.92, H_E_: 0.675; Hilsdorf & Hallerman, 2017). Considering that this is the first study performed for *Cyphocharax* genus, high diversity was found in comparison to species of the same family as *C. mivartii* (Na: 10.493, H_O_: 0.757, H_E_: 0.801; Landínez-García & Marquez, 2018) with whom it shares its habitat. Likewise, when comparing these results with species of phylogenetically related families that also inhabit the Cauca river, *C. magdalenae* displayed greater values over the two estimators with respect to *I. longirostris* (Na: 8.84 – 11. 05, H_O_: 0.701 – 0.767, H_E_: 0.771- 0.798; Landínez-García & Márquez, 2016) and similar values to those reported for *P. magdalenae* (Na: 22.455, H_O_: 0.725, H_E_: 0.898; Landínez-García et al., 2020) in the Cauca river. Even this study performed for *P. magdalenae*, which included reports of rivers with great importance for Colombia, such as San Jorge (Na: 13.545, H_O_: 0.809; H_E_: 0.884), Sinú (Na: 15.273, H_O_: 0.767, H_E_: 0.882), Atrato (Na: 14.636, H_O_: 0.718, H_E_: 0.879), Nare (Na: 15.636, H_O_: 0.659; H_E_: 0.876), and Magdalena river (Na: 19.455, H_O_: 0.758; H_E_: 0.896), *C. magdalenae* showed greater values in alleles/*locus* and similar values for H_E_. *C. magdalenae* also exhibited much greater values of H_E_ with respect to *P. reticulatus* (H_E_: 0.400) even though this estimator was calculated based on a different molecular marker (Macias et al., 2009).

Furthermore, the hypothesis of high levels of consanguinity in *C. magdalenae* was not confirmed since the species showed inbreeding signals below 10% in each genetic stock (F_IS_ Stock1=0.051; F_IS_ Stock2=0.071). Although these values do not exceed the proposed limit (Franklin, 1980; Soule, 1980), it has been indicated that any inbreeding coefficient greater than cero has unfavorable effects on the biological efficacy (Frankham et al., 2014). Considering these results and those reported for Characiformes in the Magdalena-Cauca basin using species specific microsatellites, *C. magdalenae* exhibited levels of inbreeding relatively similar to those reported for *C. mivartii* (0.040 – 0.087; Landínez-García & Marquez, 2018); lower values to those described for *P. magdalenae* (0.125 – 0.255; Landínez-García et al., 2020) and greater to those found in *Brycon henni* (-0.040 – -0.009; Landínez-García & Márquez, 2020), a phylogenetically farther species.

Additionally, this study corroborated the hypothesis of gene flow despite having found significant differences among the individuals of the lower sector of the Cauca river as said differences are related to the coexistence of two genetic stocks with uneven distribution through the river. This indicates that there is no evidence of barriers separating the gene flow of *C. magdalenae* in the low section of the Cauca river, which is consistent with data reported for *C. mivartii* (Landínez-García & Marquez, 2018), *P. magdalenae* (Landínez-García et al., 2020), *Cynopotamus magdalenae* (Steindachner 1879), *Megaleporinus muyscorum* (Steindachner 1900) (Márquez et al., 2020) and catfishes *A. pardalis*, *P. grosskopfii*, *S. cuspicaudus* (Restrepo-Escobar et al., 2021)*, P. magadaleniatum* (García-Castro et al., 2021), *P. atricaudus* and *P. magnus* (Rangel-Medrano & Márquez, 2021). Nonetheless, genetic structure due to seasonal variation was found since comparisons over the samples in rainy and drought periods during the assessed years allowed detecting that a stock predominates in the dry season (stock 1) while the other stock predominates in the rainy season (stock 2) (**Figs. 3A, 3B**). Sampling in both seasons for two years reflects an alternated cyclic behavior between both stocks and greater proliferative advantage of Stock2 over Stock1, which suggests a differential reproductive success leading to different cohorts (temporal Wahlund effect). These results are similar to those reported by Da Rosa et al., 2022 for *Prochilodus lineatus* (Valenciennes 1837) in the Mogi-Guaçu river in Brazil as the individuals analyzed between 2005 and 2006, in rainy and dry seasons (January and August, respectively) showed evidence of temporal genetic structure. Furthermore, it was found that *Salminus brasiliensis* (Cuvier 1816) in the Uruguay river in Brazil is formed by three genetic stocks resulting from a temporal genetic structure in which seasonal precipitations in the river are one of the main factors that could have originated genetic groups as the water level is fundamental in reproductive migration (Ribolli et al., 2017).

Furthermore, this study supports the hypothesis of a recent genetic bottleneck in *C. magdalenae.* These results may be a consequence of the joint action of various anthropogenic activities impacting the environment or directly affecting the *C. magdalenae* individuals, or climatic changes. For instance, for The IUCN, *C. magdalenae* shows a decrease in its populations due to the fishing pressure **(Lasso et al., 2011; Duarte et al., 2019, 2021)**, which is consistent with the reduction reports for the species in the Magdalena basin with a diminishment in 887.7 tones unloaded for 2019 (Duarte et al., 2019) in comparison to 436,1 tons for 2021 (Duarte et al., 2021). Moreover, the species does not have a recommended (R) minimum capture size nor suggested by any in force regulation (N) (AUNAP-UNIMAGDALENA, 2013); in fact, it was found that between 2018 – 2021 ranges of capture size were lower than those described for size at maturity (Duarte et al., 2021). As it has been proposed for other species (Restrepo-Escobar et al., 2021), contamination with products derived from mining, could be affecting the viability of *C. magdalenae*, which is supported by evidence of methylmercury in fishes collected in the zone (Cruz-Esquivel & Marrugo-Negrete, 2022). Another threaten is the establishment of introduced exotic species (Lasso et al., 2011), such as Basa fish *Pangasianodon hypophthalmus* (Sauvage 1878), omnivorous species of fast growth in size and weight, and extension in Colombia mainly in the Magdalena-Cauca basin (Corporación Autónoma Regional de Cundinamarca, 2019), and the Nile Tilapia *Oreochromis niloticus* (Linnaeus 1758), which has survival ability in all types of habitats, from salty, marine waters to estuarine and continental waters with temperatures between 8 and 42 °C (Corporación Autónoma Regional de Cundinamarca, 2018). Despite the introduction of *O. niloticus* in Colombia was done with commercial purposes and control of *Oreochromis mossambicus* (Peters 1852), an aggressive behavior against native species *P. magdalenae* has been displayed (Rico, 2010) and, after its introduction, there has been a reduction in the abundance of *Triportheus magdalenae* (Steindachner 1878) in the Guajaro reservoir in the Magdalena (Caraballo & Gandara, 2009)*.*

In addition to the anthropogenic activities, it has been documented that the impact of climate factors has provoked changes in fish populations as they are responsible for abrupt changes in environments such as high temperatures, alterations of hydrological cycles, reduction of dissolved oxygen, changes in mortality rates, growth, reproduction and distribution of fish populations (Serna-López & Cañón-Barriga, 2020). This climate factor has also been used for explaining population genetics aspects of *C. mivartii* (Landínez-García et al., 2016) and *P. magdalenae* (Landínez-García et al., 2020).

Contrary to the bottleneck evidence, estimations performed over the effective population size (Stock1: 1464.6, Stock2: 2127) were higher than the critical value (Ne ≤ 1000), which allows deducting that the species has long term maintenance (Frankham et al., 2014). This idea is also supported by the results of conservation status as even in 100 generations (260 years: species with generational time of 2.6 years; Puentes et al., 2014) estimations did not show evidence of reductions in heterozygosity greater than 25% (critical value of the classification), for which the species can be listed as non-threatened. This classification, however, should be interpreted carefully since it has been indicated that between 4-10% of freshwater species in South America are exposed to a certain risk of extinction mainly due to habitat loss or degradation (Reis et al., 2016).

To conclude, this study allowed determining that *C. magdalenae* in the low section of the Cauca river exhibits seasonal population structure formed by two genetic stocks associated to the rainy and dry seasons, and which show high genetic diversity, low inbreeding indexes, bottleneck signals and large effective population size. Data obtained along with the microsatellite *loci* developed *de novo* in this study are a starting point for future research directed to the monitoring of the genetic diversity and population structure of this species to develop proper management plans.

**Acknowledgements**

This study was supported by a grant framed under the Project “Variabilidad genética de un banco de peces de los sectores medio y bajo del río Cauca” (CT-2019-000661, Empresas Públicas de Medellín and Universidad Nacional de Colombia, Sede Medellín).

**References**

Aguirre-Pabón, J., J. Narváez Barandica, & L. Castro García, 2013. Mitochondrial DNA variation of the bocachico *Prochilodus magdalenae* (Characiformes, Prochilodontidae) in the Magdalena River Basin, Colombia. Aquatic Conservation: Marine and Freshwater Ecosystems 23: 594–605. https://doi.org/ 10.1002/aqc.2339.

Andrade-C., M. G., 2011. Estado del conocimiento de la biodiversidad en Colombia y sus amenazas. Consideraciones para fortalecer la interacción ciencia-política. Revista de la Academia Colombiana de Ciencias Exactas, Físicas y Naturales 35: 491–507, http://www.scielo.org.co/scielo.php?script=sci_arttext&pid=S0370-39082011000400008&lng=es&nrm=iso&tlng=es.

AUNAP-UNIMAGDALENA, 2013. Tallas mínimas de captura para el aprovechamiento sostenible de las principales especies de peces, crustáceos y moluscos comerciales de Colombia. pp. 58. http://sepec.unimagdalena.edu.co/Archivos/Cartilla - TALLAS MINIMAS_Digital (2).pdf.

Berdugo, G. O., & J. C. N. Barandica, 2014. Genetic diversity and population structure of bocachico *Prochilodus magdalenae* (Pisces, Prochilodontidae) in the Magdalena river basin and its tributaries, Colombia. Genetics and Molecular Biology 37: 37–45. https://doi.org/ 10.1590/S1415-47572014000100008.

Blacket, M. J., C. Robin, R. T. Good, S. F. Lee, & A. D. Miller, 2012. Universal primers for fluorescent labelling of PCR fragments-an efficient and cost-effective approach to genotyping by fluorescence. Molecular Ecology Resources 12: 456–463. https://doi.org/ 10.1111/j.1755-0998.2011.03104.x.

Blanco, H. V, J. Solipá, C. W. Olaya-Nieto, F. F. Segura-Guevara, S. B. Brú-Cordero, & G. Tordecilla-Petro, 2005. Crecimiento y mortalidad de la yalua (*Cyphocharax magdalenae* steindachner, 1878) en el río Sinú, Colombia. Revista MVZ Córdoba 10: 555–563, http://www.scielo.org.co/scielo.php?script=sci_arttext&pid=S0122-02682005000100005&lang=es%0Ahttp://www.scielo.org.co/pdf/mvz/v10n1/v10n1a05.pdf.

Bostein, D., R. L. White, M. Skolnick, & R. W. Davis, 1980. Construction of a genetic linkage map in man using restriction fragment length polymorphisms. American Journal of Human Genetics 32: 314–331. https://www.ncbi.nlm.nih.gov/pmc/articles/PMC1686077/pdf/ajhg00189-0020.pdf

Caraballo, P., & M. Gandara, 2009. El sector pesquero en el departamento de sucre, Colombia. Rev. Colombiana cienc. Anim. 1: 273–278. https://dialnet.unirioja.es/servlet/articulo?codigo=3268849

Carvajal, Y., A. Enciso, & M. Sandoval, 2016. Hydrological analysis of historical floods in the upper valley of Cauca river. Ingeniería y Competitividad 18: 46. https://doi.org/ 10.25100/iyc.v18i1.2176.

Castoe, T. A., A. W. Poole, W. Gu, A. P. Jason de Koning, J. M. Daza, E. N. Smith, & D. D. Pollock, 2010. Rapid identification of thousands of copperhead snake (*Agkistrodon contortrix*) microsatellite *loci* from modest amounts of 454 shotgun genome sequence. Molecular Ecology Resources 10: 341–347. https://doi.org/10.1111/j.1755-0998.2009.02750.x.

Cerón, W. L., M. T. Kayano, C. Ocampo-Marulanda, T. Canchala, I. A. Rivera, A. Avila-Diaz, R. V. Andreoli, & I. P. de Souza, 2021. Spatio-temporal variability of hydroclimatology in the upper Cauca river basin in southwestern Colombia: Pre-and post-Salvajina dam perspective. Atmosphere 12. https://doi.org/ 10.3390/atmos12111527.

Corporación Autónoma Regional de Cundinamarca, 2018. Plan de prevención, control y manejo de la Tilapia del Nilo (*Oreochromis niloticus*) en la jurisdicción CAR Cundinamarca. pp. 34. https://www.car.gov.co/uploads/files/5b90332505307.pdf

Corporación Autónoma Regional de Cundinamarca, 2019. Plan de Prevención, Control y Manejo (PPCM) del pez basa *(Pangasianodon hypophthalmus*) para la jurisdicción de la Corporación Autónoma Regional de Cundinamarca CAR. pp. 56. https://www.car.gov.co/uploads/files/5ef5299cb43ad.pdf

Cruz-Esquivel, Á., & J. Marrugo-Negrete, 2022. Concentraciones de metilmercurio en *Prochilodus magdalenae* (Teleostei: Curimatidae) y *Hoplias malabaricus* (Teleostei: Erythrinidae) en la cuenca baja del río Cauca-Magdalena, norte de Colombia. Acta Biologica Colombiana 27: 28–35. https://doi.org/ 10.15446/abc.v27n1.83092.

Da Rosa, I. F., D. Deoliveira, V. Dacruz, F. Ashikaga, G. Costa, L. Doretto, J. Senhorini, C. R. Ritade, F. Foresti, C. Oliveira, & F. Foresti, 2022. Temporal genetic structure of a stock of *Prochilodus lineatus* (Characiformes: Prochilodontidae) in the Mogi-Guaçu River ecosystem, São Paulo, Brazil. Neotropical Ichthyology 20: 1–18. https://doi.org/ 10.1590/1982-0224-2021-0156.

Do, C., R. S. Waples, D. Peel, G. M. Macbeth, B. J. Tillett, & J. R. Ovenden, 2014. NeEstimator v2: Re-implementation of software for the estimation of contemporary effective population size (Ne) from genetic data. Molecular Ecology Resources 14: 209–214. https://doi.org/10.1111/1755-0998.12157.

DoNascimiento, C., J. D. Bogotá Gregory, J. G. Albornoz Garzón, A. Mendez López, F. A. Villa-navarro, E. E. Herrera-Collazos, H. Agudelo Zamora, M. Arce H., & Geles, 2023. Lista de especies de peces de agua dulce de Colombia / Checklist of the freshwater fishes of Colombia. v2.15. https://doi.org/10.15472/numrso

DoNascimiento, C., E. E. Herrera-Collazos, G. A. Herrera-R, A. Ortega-Lara, F. A. Villa-Navarro, J. S. U. Oviedo, & J. A. Maldonado-Ocampo, 2017. Checklist of the freshwater fishes of Colombia: A Darwin core alternative to the updating problem. ZooKeys 708: 25–138. https://doi.org/ 10.3897/zookeys.708.13897.

Duarte, L. O., C. Cuervo, O. Vargas, B. Gil-Manrique, F. Cuello, G. De León, E. Isaza, K. Tejeda, L. Manjarrés–Martínez, & H. Reyes-Ardila, 2021. Estadísticas de desembarco y esfuerzo de las pesquerías artesanales de Colombia 2021. Informe técnico. Autoridad Nacional de Acuicultura y Pesca (AUNAP), Universidad del Magdalena. Santa Marta. pp. 169. http://sepec.aunap.gov.co/Archivos/Boletines-2021/SEPEC_Boletin_Desembarco_Artesanal_2021.pdf

Duarte, L. O., L. Manjarrés–Martínez, & H. Reyes-Ardila, 2019. Estadísticas de desembarco y esfuerzo de las pesquerías artesanales e industriales de Colombia entre febrero y diciembre de 2019. Autoridad Nacional de Acuicultura y Pesca (AUNAP). Bogota. pp. 95. http://sepec.aunap.gov.co/Archivos/Boletines-2019/Boletin_Pesca_Consumo_artesanal_industrial_2019.pdf

Evanno, G., S. Regnaut, & J. Goudet, 2005. Detecting the number of clusters of individuals using the software STRUCTURE: A simulation study. Molecular Ecology 14: 2611–2620. https://doi.org/ 10.1111/j.1365-294X.2005.02553.x.

Excoffier, L., G. Laval, & S. Schneider, 2005. Arlequin (version 3.0): An integrated software package for population genetics data analysis. Evolutionary Bioinformatics 1: 117693430500100. https://doi.org/ 10.1177/117693430500100003.

Fernandez-Silva, I., J. Whitney, B. Wainwright, K. R. Andrews, H. Ylitalo-Ward, B. W. Bowen, R. J. Toonen, E. Goetze, & S. A. Karl, 2013. Microsatellites for Next-Generation Ecologists: A Post-Sequencing Bioinformatics Pipeline. PLoS ONE 8: e55990. https://doi.org/ 10.1371/journal.pone.0055990.

Foll, M., & O. Gaggiotti, 2008. A genome-scan method to identify selected *loci* appropriate for both dominant and codominant markers: A Bayesian perspective. Genetics 180: 977–993. https://doi.org/ 10.1534/genetics.108.092221.

Frankham, R., C. J. A. Bradshaw, & B. W. Brook, 2014. Genetics in conservation management: Revised recommendations for the 50/500 rules, Red List criteria and population viability analyses. Biological Conservation Elsevier Ltd 170: 56–63. https://doi.org/ 10.1016/j.biocon.2013.12.036.

Franklin, I. R., 1980. Evolutionary changes in small populations En Soulé, M., & B. Wilcox (eds), Conservation biology: an evolutionary-ecological prospective. Sinauer Associates, Sunderland: 135–149.

Fricke, R., W. N. Eschmeyer, & R. Van Der Laan, 2022. Eschmeyer’s catalog of fishes: genera, species, references. http://researcharchive.calacademy.org/research/ichthyology/catalog/SpeciesByFamily.asp.

Gallo, H., & J. Díaz-sarmiento, 2003. Variabilidad genética del bagre *rayado Pseudoplatystoma fasciatum* (Pisces: Pimelodidae) en el río Magdalena. Rev. Acad. Colomb. Cienc. 105: 599–605.

García-Castro, K. L., J. D. Rangel-Medrano, R. M. Landínez-García, & E. J. Márquez, 2021. Population genetics of the endangered catfish *Pseudoplatystoma magdaleniatum* (Siluriformes: Pimelodidae) based on species-specific microsatellite *loci*. Neotropical Ichthyology 19: 1–15. https://doi.org/ 10.1590/1982-0224-2020-0120.

Garza, J. C., & E. G. Williamson, 2001. Detection of reduction in population size using data from microsatellite *loci*. Molecular Ecology 10: 305–318.

González-López, N., & Y. Carvajal-Escobar, 2020. Characterization of hydrological drought in the Cauca river high valley. Tecnologia y Ciencias del Agua 11: 235–264. https://doi.org/10.24850/j-tyca-2020-01-06.

Hilsdorf, A. W. S., & E. M. Hallerman, 2017. Genetic resources of neotropical fishes. Genetic Resources of Neotropical Fishes. Springer International Publishing, Switzerland. https://doi.org/ 10.1007/978-3-319-55838-7.

Holm, S., 1979. A simple sequentially rejective multiple test procedure. Scandinavian Journal of Statistics 6: 65–70. http://www.jstor.org/stable/4615733%0Ahttp://www.jstor.org/page/info/about/policies/terms.jsp%0Ahttp://www.jstor.org.

Hurtado-Alarcón, J. C., N. J. Mancera-Rodriguez, & C. I. Saldamando-Benjumea, 2011. Variabilidad genética de *Brycon henni* (Characiformes: Characidae) en la cuenca media de los ríos Nare y Guatapé, sistema Río Magdalena, Colombia. Revista de Biología Tropical 59: 269–282.

Jimenéz-Segura, L. & C. A. Lasso (Eds.). 2020. XIX. Peces de la cuenca del río Magdalena, Colombia: diversidad, conservación y uso sostenible. Serie Editorial Recursos Hidrobiológicos y Pesqueros Continentales de Colombia. 434 pp. https://doi.org/ 10.21068/a2020rrhhxix.

Jombart, T., 2008. Adegenet: A R package for the multivariate analysis of genetic markers. Bioinformatics 24: 1403–1405. https://doi.org/ 10.1093/bioinformatics/btn129.

Jost, L., 2008. GST and its relatives do not measure differentiation. Molecular Ecology 17: 4015–4026. https://doi.org/ 10.1111/j.1365-294X.2008.03887.x.

Joya, C. D., R. M. Landínez-García, & E. J. Márquez, 2021. Development of microsatellite *loci* and population genetics of the catfish *Pimelodus yuma* (Siluriformes: Pimelodidae). Neotropical Ichthyology 19: 1–15. https://doi.org/ 10.1590/1982-0224-2020-0114.

Kopelman, N. M., J. Mayzel, M. Jakobsson, N. A. Rosenberg, & I. Mayrose, 2015. CLUMPAK : a program for identifying clustering modes and packaging population structure inferences across K. Mol Ecol Resour. 15: 1179–1191. https://doi.org/ 10.1111/1755-0998.12387.

Landínez-García, R. M., J. F. Alzate, & E. J. Márquez, 2016. Mitochondrial genome of the Neotropical detritivore fish *Curimata mivartii* Steindachner 1878 (Characiformes, Curimatidae). Mitochondrial DNA. Part A, DNA mapping, sequencing, and analysis 27: 2196–2197. https://doi.org/ 10.3109/19401736.2014.982623.

Landínez-García, R. M., & E. J. Marquez, 2016. Development and characterization of 24 polymorphic microsatellite *loci* for the freshwater fish *Ichthyoelephas longirostris* (Characiformes: Prochilodontidae). PeerJ 4: e2419. https://doi.org/ 10.7717/peerj.2419.

Landínez-García, R. M., & E. J. Marquez, 2018. Microsatellite *loci* development and population genetics in neotropical fish *Curimata mivartii* (Characiformes: Curimatidae). PeerJ 12–17. https://doi.org/ 10.7717/peerj.5959.

Landínez-García, R. M., & E. J. Márquez, 2020. Population genetics of the fish *Brycon henni* (Characiformes: Bryconidae) using species-specific polymorphic microsatellite *loci*. Revista de Biología Tropical 68: 847–861. https://doi.org/ 10.15517/RBT.V68I3.38405.

Landínez-García, R. M., J. C. Narváez, & E. J. Márquez, 2020. Population genetics of the freshwater fish *Prochilodus magdalenae* (Characiformes: Prochilodontidae), using species-specific microsatellite *loci*. PeerJ 1–26. https://doi.org/ 10.7717/peerj.10327.

Lasso, C. A., F. de P. Gutiérrez, M. A. Morales-Betancourt, E. Agudelo, H. Ramírez-Gil, & R. E. Ajiaco-Martínez (eds), 2011. II. Pesquerías continentales de Colombia: cuencas del Magdalena-Cauca, Sinú, Canalete, Atrato, Orinoco. Instituto de Investigación de los Recursos Biológicos Alexander von Humboldt, Bogota, http://hdl.handle.net/20.500.12324/19395.

Li, Y.-L., & J.-X. Liu, 2018. StructureSelector: a web based software to select and visualize the optimal number of clusters by using multiple methods. Mol Ecol Resour. 18: 176–177. https://doi.org/ 10.1111/1755-0998.12719.

Luikart, G., & J. M. Cornuet, 1998. Empirical evaluation of a test for identifying recently bottlenecked populations from allele frequency data. Conservation Biology 12: 228–237. https://doi.org/ 10.1046/j.1523-1739.1998.96388.x.

Macias, J. N. L., F. G. Vallejo, E. R. Rincón, A. C. Giraldo, & F. Cerón, 2009. Diversidad genética del bocachico (*Prochilodus reticulatus*) de la cuenca alta del río Cauca (Colombia). Acta Biológica Paranaense 38: 113-138.https://doi.org/ 10.5380/abpr.v38i0.16928.

Márquez, E. J., N. Restrepo-Escobar, A. J. Yepes-Acevedo, & J. C. Narváez-Barandica, 2020. Diversidad y estructura genética de los peces de la cuenca del Río Magdalena, Colombia En Jiménez-Segura, L., & C. A. Lasso (eds), Peces de la cuenca del río Magdalena, Colombia: diversidad, conservación y uso sostenible. Serie Editorial Recursos Hidrobiológicos y Pesqueros Continentales de Colombia. Instituto de Investigación de Recursos Biológicos Alexander von Humboldt: 115–157. https://doi.org/ 10.21068/a2020rrhhxix.

Marshall, T. C., J. Slate, L. E. B. Kruuk, & J. M. Pemberton, 1998. Statistical confidence for likelihood-based paternity inference in natural populations. Molecular Ecology 7: 639–655. https://doi.org/ 10.1046/j.1365-294x.1998.00374.x.

Meirmans, P. G., 2006. Using the Amova framework to estimate a standardized genetic differentiation measure. Evolution 60: 2399–2402. https://doi.org/ 10.1554/05-631.1

Meirmans, P. G., & P. W. Hedrick, 2011. Assessing population structure: FST and related measures. Molecular Ecology Resources 11: 5–18. https://doi.org/ 10.1111/j.1755-0998.2010.02927.x.

Peakall, R., & P. E. Smouse, 2006. GENALEX 6: Genetic analysis in Excel. Population genetic software for teaching and research. Molecular Ecology Notes 6: 288–295. https://doi.org/ 10.1111/j.1471-8286.2005.01155.x

Pineda, H., L. Arboleda, A. Echeverry, S. Urcuqui, D. Pareja, M. Oliveria, & J. Builes, 2007. Caracterización de la diversidad genética en el pez *Brycon henni* (Characiformes: Characidae) en Colombia central por medio de marcadores RAPD. Revista de Biología Tropical 55: 1025–1035.

Piry, S., G. Luikart, & J. M. Cornuet, 1999. BOTTLENECK: A computer program for detecting recent reductions in the effective population size using allele frequency data. Journal of Heredity 90: 502–503. https://doi.org/ 10.1093/jhered/90.4.502.

Pritchard, J. K., M. Stephens, & P. Donnelly, 2000. Inference of population structure using multilocus genotype data. Genetics 155: 945–959. https://doi.org/10.1093/genetics/155.2.945

Puechmaille, S. J., 2016. The program structure does not reliably recover the correct population structure when sampling is uneven: Subsampling and new estimators alleviate the problem. Molecular Ecology Resources 16: 608–627. https://doi.org/ 10.1111/1755-0998.12512.

Rangel-Medrano, J. D., & E. J. Márquez, 2021. Development of microsatellite *loci* and population genetics in the bumblebee catfish species *Pseudopimelodus atricaudus* and *Pseudopimelodus magnus* (Siluriformes: Pseudopimelodidae). Neotropical Ichthyology 19: 1–15. https://doi.org/ 10.1590/1982-0224-2020-0053.

Restrepo-Escobar, N., & E. J. Márquez, 2020. Microsatellite *loci* development for three catfish species from Northwestern South America. Neotropical Ichthyology 18: 1–16. https://doi.org/ 10.1590/1982-0224-2019-0079.

Restrepo-Escobar, N., A. J. Yepes-Acevedo, & E. J. Márquez, 2021. Population genetics of three threatened catfish species in heterogeneous environments of the Cauca river, Colombia. Neotropical Ichthyology 19: 1–18. https://doi.org/ 10.1111/jfb.13016.

Ribolli, J., D. J. Hoeinghaus, J. A. Johnson, E. Zaniboni-Filho, P. D. de Freitas, & P. M. Galetti, 2017. Isolation-by-time population structure in potamodromous Dourado *Salminus brasiliensis* in southern Brazil. Conservation Genetics Springer Netherlands 18: 67–76. https://doi.org/ 10.1007/s10592-016-0882-x.

Rice, W. R., 1989. Analyzing Tables of Statistical Tests. Evolution 43: 223–225. https://doi.org/ 10.1007/978-1-4613-8622-3_27.

Rico, G., 2010. Fauna exótica e invasora en Colombia. Instituto de Investigación de Recursos Biológicos Alexander von Humboldt. Retrieved from http://repository.humboldt.org.co/bitstream/handle/20.500.11761/31181/10-049.pdf?sequence=1&isAllowed=y

Rotmistrovsky, K., W. Jang, & G. D. Schuler, 2004. A web server for performing electronic PCR. Nucleic Acids Research 32: 108–112. https://doi.org/ 10.1093/nar/gkh450.

Rozen, S., & H. Skaletsky, 2000. Primer3 on the WWW for general users and for biologist programmers. Bioinformatics Methods and Protocols 132: 365–386. https://doi.org/ 10.1385/1-59259-192-2:365.

Schoebel, C. N., S. Brodbeck, D. Buehler, C. Cornejo, J. Gajurel, H. Hartikainen, D. Keller, M. Leys, Š. Říčanová, G. Segelbacher, S. Werth, & D. Csencsics, 2013. Lessons learned from microsatellite development for nonmodel organisms using 454 pyrosequencing. Journal of Evolutionary Biology 26: 600–611. https://doi.org/ 10.1111/jeb.12077.

Serna-lópez, J. P., & J. E. Cañón-barriga, 2020. Projecting the future of ayapel cienaga: A hydroecologic analysis under climate change scenarios. Revista Facultad de Ingenieria 95: 73–87. https://doi.org/ 10.17533/udea.redin.20190940.

Serna, J. C., Grupo de Modelamiento de Tiempo y Clima, F. Bernal, L. M. Moreno, V. Camacho, & N. Traslaviña, 2021. Boletín de predicción climática y recomendación sectorial: Para planear y decidir. Retrieved from http://www.ideam.gov.co/documents/21021/112700454/12_Bolet%C3%ADn_Predicci%C3%B3n_Clim%C3%A1tica_Diciembre_2021.pdf/3e09488d-8aee-459b-8a20-00ab84983279?version=1.0

Soule, M. E., 1980. Thresholds for Survival: Maintaining Fitness and Evolutionary Potential En Soulé, M., & B. Wilcox (eds), Conservation Biology: an Evolutionary-Ecological Perspective. Sinauer Associates, Sunderland: 151–169.

Van Oosterhout, C., W. F. Hutchinson, D. P. M. Wills, & P. Shipley, 2004. MICRO-CHECKER: Software for identifying and correcting genotyping errors in microsatellite data. Molecular Ecology Notes 4: 535–538. https://doi.org/ 10.1111/j.1471-8286.2004.00684.x.

Wright, S., 1943. Isolation by distance. Genetics 28: 114-138. https://doi.org/ 10.1109/IGARSS.2006.90.

Wright, S., 1965. The interpretation of population structure by F-statistics with special regard to systems of mating. Evolution 19: 395–420. https://doi.org/10.2307/2406450

Zapata, L. A., & J. S. Usma, 2013. Guía de las especies migratorias de la biodiversidad en Colombia. Plan Nacional de las Especies Migratorias. Retrieved from https://www.minambiente.gov.co/wp-content/uploads/2021/10/Gui%CC%81a-especies-migratorias-de-la-biodiversidad-en-Colombia-Volumen-2-peces.pdf
