# Supplementary material for: Genetic insights into Cyphocharax magdalenae (Characiformes: Curimatidae): Microsatellite loci development and population analysis in the Cauca River, Colombia
Source: PLoS One. 2024 Apr 16;19(4):e0302273. doi: 10.1371/journal.pone.0302273 (PMC11020439; doi:10.1371/journal.pone.0302273)
Supplement: Attachment — Submitted filename: PONE_Cyphocharax_Response_to_Reviewers.docx [file pone.0302273.s005.docx]

Medellín, February 9th, 2024

Dr. Emily Chenette

Editor-in-chief PLOS ONE

Dear Editor

We really appreciate the detailed revision of the reviewers and have edited the manuscript following your valuable recommendations, which have led to improve our paper.

We hope that the manuscript is now suitable for publication in *Plos One.*

Sincerely,

Edna J. Márquez

PONE-D-23-37212

Genetic insights into *Cyphocharax magdalenae* (Characiformes: Curimatidae): microsatellite loci development and population analysis in the Cauca River, Colombia

PLOS ONE

Dear Dr. Marquez,

Thank you for submitting your manuscript to PLOS ONE. After careful consideration, we feel that it has merit but does not fully meet PLOS ONE’s publication criteria as it currently stands. Therefore, we invite you to submit a revised version of the manuscript that addresses the points raised during the review process.

ACADEMIC EDITOR:

Please consider the following comments while revising your submission:

Some sentences are too long, which make the communication unclear.

Done

The manuscript needs to be proofread for English language and grammatical fixes by a proficient speaker.

Done.

Other suggestions are indicated in the attached manuscript file.

Please submit your revised manuscript by Feb 09 2024 11:59PM. If you will need more time than this to complete your revisions, please reply to this message or contact the journal office at plosone@plos.org. When you're ready to submit your revision, log on to https://www.editorialmanager.com/pone/ and select the 'Submissions Needing Revision' folder to locate your manuscript file.

Please include the following items when submitting your revised manuscript:

A rebuttal letter that responds to each point raised by the academic editor and reviewer(s). You should upload this letter as a separate file labeled 'Response to Reviewers'.

Done

A marked-up copy of your manuscript that highlights changes made to the original version. You should upload this as a separate file labeled 'Revised Manuscript with Track Changes'.

Done

An unmarked version of your revised paper without tracked changes. You should upload this as a separate file labeled 'Manuscript'.

Done

If you would like to make changes to your financial disclosure, please include your updated statement in your cover letter. Guidelines for resubmitting your figure files are available below the reviewer comments at the end of this letter.

Please see below.

If applicable, we recommend that you deposit your laboratory protocols in protocols.io to enhance the reproducibility of your results. Protocols.io assigns your protocol its own identifier (DOI) so that it can be cited independently in the future. For instructions see: https://journals.plos.org/plosone/s/submission-guidelines#loc-laboratory-protocols. Additionally, PLOS ONE offers an option for publishing peer-reviewed Lab Protocol articles, which describe protocols hosted on protocols.io. Read more information on sharing protocols at https://plos.org/protocols?utm_medium=editorial-email&utm_source=authorletters&utm_campaign=protocols.

Complete information is documented in the manuscript.

We look forward to receiving your revised manuscript.

Kind regards,

Timothy Omara, PhD

Academic Editor

PLOS ONE

Journal requirements:

1. When submitting your revision, we need you to address these additional requirements.

Please ensure that your manuscript meets PLOS ONE's style requirements, including those for file naming. The PLOS ONE style templates can be found at

https://journals.plos.org/plosone/s/file?id=wjVg/PLOSOne_formatting_sample_main_body.pdf and

https://journals.plos.org/plosone/s/file?id=ba62/PLOSOne_formatting_sample_title_authors_affiliations.pdf.

Done

2. Thank you for stating the following in the Acknowledgments Section of your manuscript:

[This study was supported by a grant framed under the Project “Variabilidad genética de un banco de peces de los sectores medio y bajo del río Cauca” (CT-2019-000661, Empresas Públicas de Medellín and Universidad Nacional de Colombia, Sede Medellín).]

We note that you have provided funding information that is not currently declared in your Funding Statement. However, funding information should not appear in the Acknowledgments section or other areas of your manuscript. We will only publish funding information present in the Funding Statement section of the online submission form.

Please remove any funding-related text from the manuscript and let us know how you would like to update your Funding Statement. Currently, your Funding Statement reads as follows:

[This study was supported by a grant framed under the Project “Variabilidad genética de un banco de peces de los sectores medio y bajo del río Cauca” (CT-2019-000661, Empresas Públicas de Medellín and Universidad Nacional de Colombia, Sede Medellín). Funders do not play any role in the study design, data collection and analysis, decision to publish, or preparation of the manuscript]

Please include your amended statements within your cover letter; we will change the online submission form on your behalf.

Done. We have removed the text, and we would like to retain the current Funding Statement.

3. Please confirm at this time whether or not your submission contains all raw data required to replicate the results of your study. Authors must share the “minimal data set” for their submission. PLOS defines the minimal data set to consist of the data required to replicate all study findings reported in the article, as well as related metadata and methods (https://journals.plos.org/plosone/s/data-availability#loc-minimal-data-set-definition).

For example, authors should submit the following data:

- The values behind the means, standard deviations and other measures reported;

- The values used to build graphs;

- The points extracted from images for analysis.

Authors do not need to submit their entire data set if only a portion of the data was used in the reported study.

If your submission does not contain these data, please either upload them as Supporting Information files or deposit them to a stable, public repository and provide us with the relevant URLs, DOIs, or accession numbers. For a list of recommended repositories, please see <https://journals.plos.org/plosone/s/recommended-repositories>.

Now, we included the Supporting information “S1 Table”: “S1 Table. Genotype data at 14 microsatellite loci included in the population genetic analysis of *Cyphocharax magdalenae*. First row indicates respectively: Number of loci, Number of individuals, Number of sampling sections, Sample size for five sections. Third row indicates respectively: Sample ID, Sampling section, Genetic stock, Locus name for 14 loci.”

If there are ethical or legal restrictions on sharing a de-identified data set, please explain them in detail (e.g., data contain potentially sensitive information, data are owned by a third-party organization, etc.) and who has imposed them (e.g., an ethics committee). Please also provide contact information for a data access committee, ethics committee, or other institutional body to which data requests may be sent. If data are owned by a third party, please indicate how others may request data access.

4. We note that Figure 1 in your submission contain [map/satellite] images which may be copyrighted. All PLOS content is published under the Creative Commons Attribution License (CC BY 4.0), which means that the manuscript, images, and Supporting Information files will be freely available online, and any third party is permitted to access, download, copy, distribute, and use these materials in any way, even commercially, with proper attribution. For these reasons, we cannot publish previously copyrighted maps or satellite images created using proprietary data, such as Google software (Google Maps, Street View, and Earth). For more information, see our copyright guidelines: http://journals.plos.org/plosone/s/licenses-and-copyright.

We require you to either (1) present written permission from the copyright holder to publish these figures specifically under the CC BY 4.0 license, or (2) remove the figures from your submission:

a. You may seek permission from the original copyright holder of Figure 1 to publish the content specifically under the CC BY 4.0 license.

We recommend that you contact the original copyright holder with the Content Permission Form (http://journals.plos.org/plosone/s/file?id=7c09/content-permission-form.pdf) and the following text:

“I request permission for the open-access journal PLOS ONE to publish XXX under the Creative Commons Attribution License (CCAL) CC BY 4.0 (http://creativecommons.org/licenses/by/4.0/). Please be aware that this license allows unrestricted use and distribution, even commercially, by third parties. Please reply and provide explicit written permission to publish XXX under a CC BY license and complete the attached form.”

Please upload the completed Content Permission Form or other proof of granted permissions as an ""Other"" file with your submission.

In the figure caption of the copyrighted figure, please include the following text: “Reprinted from [ref] under a CC BY license, with permission from [name of publisher], original copyright [original copyright year].”

b. If you are unable to obtain permission from the original copyright holder to publish these figures under the CC BY 4.0 license or if the copyright holder’s requirements are incompatible with the CC BY 4.0 license, please either i) remove the figure or ii) supply a replacement figure that complies with the CC BY 4.0 license. Please check copyright information on all replacement figures and update the figure caption with source information. If applicable, please specify in the figure caption text when a figure is similar but not identical to the original image and is therefore for illustrative purposes only.

The following resources for replacing copyrighted map figures may be helpful:

USGS National Map Viewer (public domain): http://viewer.nationalmap.gov/viewer/

The Gateway to Astronaut Photography of Earth (public domain): http://eol.jsc.nasa.gov/sseop/clickmap/

Maps at the CIA (public domain): https://www.cia.gov/library/publications/the-world-factbook/index.html and https://www.cia.gov/library/publications/cia-maps-publications/index.html

NASA Earth Observatory (public domain): http://earthobservatory.nasa.gov/

Landsat: http://landsat.visibleearth.nasa.gov/

USGS EROS (Earth Resources Observatory and Science (EROS) Center) (public domain): http://eros.usgs.gov/#

Natural Earth (public domain): http://www.naturalearthdata.com/

We include a new figure: “Fig 2. Sampling sites (stars) of *Cyphocharax magdalenae* in the lower section of the Cauca River (S4-S8). Self-made creation of the map based on contour lines scaled 1:100,000 from the Instituto Geográfico Agustín Codazzi source, 2019 (Available from: IGAC Geoportal, https://geoportal.igac.gov.co/contenido/datos-abiertos-cartografia-y-geografia).”

Additional Editor Comments:

[Note: HTML markup is below. Please do not edit.]

Reviewers' comments:

Reviewer's Responses to Questions

Comments to the Author

1. Is the manuscript technically sound, and do the data support the conclusions?

The manuscript must describe a technically sound piece of scientific research with data that supports the conclusions. Experiments must have been conducted rigorously, with appropriate controls, replication, and sample sizes. The conclusions must be drawn appropriately based on the data presented.

Reviewer #1: Yes

Reviewer #2: Yes

2. Has the statistical analysis been performed appropriately and rigorously?

Reviewer #1: Yes

Reviewer #2: Yes

3. Have the authors made all data underlying the findings in their manuscript fully available?

The PLOS Data policy requires authors to make all data underlying the findings described in their manuscript fully available without restriction, with rare exception (please refer to the Data Availability Statement in the manuscript PDF file). The data should be provided as part of the manuscript or its supporting information, or deposited to a public repository. For example, in addition to summary statistics, the data points behind means, medians and variance measures should be available. If there are restrictions on publicly sharing data—e.g. participant privacy or use of data from a third party—those must be specified.

Reviewer #1: Yes

Reviewer #2: Yes

4. Is the manuscript presented in an intelligible fashion and written in standard English?

PLOS ONE does not copyedit accepted manuscripts, so the language in submitted articles must be clear, correct, and unambiguous. Any typographical or grammatical errors should be corrected at revision, so please note any specific errors here.

Reviewer #1: Yes

Reviewer #2: Yes

5. Review Comments to the Author

Please use the space provided to explain your answers to the questions above. You may also include additional comments for the author, including concerns about dual publication, research ethics, or publication ethics. (Please upload your review as an attachment if it exceeds 20,000 characters)

Reviewer #1: This paper aimed to develop the microsatellite marker and assessed the population structure of Cyphocharax magdalenae in Columbia. Authors did a great job and clearly interpret the data. However, this manuscript is too long and need to restructure as I suggested in the attached.

Done

I did not find the results of AMOVA among the population of S4-S8. Did you calculate this AMOVA?

An AMOVA using all samples from each sector was performed to assess the genetic differentiation among all sections (see Summary AMOVA Table). The pair-wise comparisons, using the statistic F´_ST_ and Jost´D_st_ are shown in the Table 5.

Summary AMOVA Table

Source	df	SS	MS	Est. Var.	%

Among Sections	4	37.244	9.311	0.020	0%

Among Indiv	319	2148.416	6.735	0.537	9%

Within Indiv	324	1834.000	5.660	5.660	91%

Total	647	4019.660	6.218	100%

This information is summarized in the text: “…with results obtained in AMOVA (F´_ST (4, 647)_ = 0.003, P = 0.001) and the pairwise comparisons of the standardized statistics F’_ST_, D_EST_ (Table 5)”

I also found something weird. You mention high genetic diversity and a recent genetic bottleneck. Bottleneck normally reduced genetic diversity. Why? I did not see you explain about this.

Now, in the third line of the penultimate paragraph: “In conjunction with the previously mentioned high genetic diversity levels, this result suggests that *C. magdalenae* populations are large enough to sustain elevated genetic diversity and Ne, despite exhibiting signals of bottleneck.”

Cheers

Reviewer #2: This paper describes a routine process of developing microsatellite marker using NGS in Cyphocharax magdalenae, which has potential to become a baseline data for further studies.

Author may explain, why novel set of markers was developed in this study? Now a days there are plenty of the records of cross-species amplification. Was crass-amplification using other closely related species' microsatellite maker failed?

Now: “**To test these hypotheses, this study identified de novo and characterized species-specific microsatellite loci to *C. magdalenae*. This approach was chosen instead of using heterologous loci to avoid potential pitfalls associated with cross-amplification [38], such as allele size homoplasy [39], unsuccessful amplification in phylogenetically distant species [40], low polymorphism, presence of null alleles [41], and amplification of non-orthologous loci [42]”**

Though there were some discussion for remaining 16 markers. However, a supplementary file may be added explaining the statistics of remaining 16 loci.

Done. Now, we included the Supporting information “S2 Table”. “S2 Table. Microsatellite loci not selected due to pitfalls in amplification or low levels of polymorphisms. Na number of alleles per locus; H_O_ Observed heterozygosity; H_E_ Expected heterozygosity.”

A reference image of study species may be provided in paper.

Done. Now, we included a photography and the respective copyright permissions: “Fig 1. Photography of *Cyphocharax magdalenae*, a neotropical freshwater fish. Courtesy of Jorge E. García-Melo and Luis J. García-Melo, Proyecto CaVFish Colombia.”

Overall this is a crisp and clear methodology paper with basic population genetics of Cyphocharax magdalenae.

6. PLOS authors have the option to publish the peer review history of their article (what does this mean?). If published, this will include your full peer review and any attached files.

If you choose “no”, your identity will remain anonymous but your review may still be made public.

Do you want your identity to be public for this peer review? For information about this choice, including consent withdrawal, please see our Privacy Policy.

Reviewer #1: No

Reviewer #2: Yes: SANDEEP KUMAR GUPTA

[NOTE: If reviewer comments were submitted as an attachment file, they will be attached to this email and accessible via the submission site. Please log into your account, locate the manuscript record, and check for the action link "View Attachments". If this link does not appear, there are no attachment files.]

While revising your submission, please upload your figure files to the Preflight Analysis and Conversion Engine (PACE) digital diagnostic tool, https://pacev2.apexcovantage.com/. PACE helps ensure that figures meet PLOS requirements. To use PACE, you must first register as a user. Registration is free. Then, login and navigate to the UPLOAD tab, where you will find detailed instructions on how to use the tool. If you encounter any issues or have any questions when using PACE, please email PLOS at figures@plos.org. Please note that Supporting Information files do not need this step.
